# Co-regulation and synteny of GFM2 and NSA2 links ribosomal function in mitochondria and the cytosol with chronic kidney disease

**DOI:** 10.1186/s10020-024-00930-8

**Published:** 2024-10-13

**Authors:** Minjie Zhang, Christer Hogstrand, Paola Pontrelli, Afshan N Malik

**Affiliations:** 1 Diabetes & Obesity, School of Cardiovascular Medicine and Metabolic Sciences, King’s College London, London, SE1 1UL UK; 2 Analytical, Environmental and Forensic Sciences, School of Cancer and Pharmaceutical Sciences, King’s College London, London, SE1 8NH UK; 3 Department of Precision and Regenerative Medicine and Ionian Area (DIMEPRE-J), University of Bari Aldo Moro, Bari, Italy

**Keywords:** NSA2, Ribosome biogenesis, Protein synthesis, Mitochondria, Gene expression, diabetic nephropathy, chronic kidney disease, mitochondrial dysfunction

## Abstract

**Background:**

We previously reported aberrant expression of the cytosolic ribosomal biogenesis factor Nop-7-associated 2 (NSA2) in diabetic nephropathy, the latter also known to involve mitochondrial dysfunction, however the connections between NSA2, mitochondria and renal disease were unclear. In the current paper, we show that NSA2 expression is co-regulated with the GTP-dependent ribosome recycling factor mitochondrial 2 (GFM2) and provide a molecular link between cytosolic and mitochondrial ribosomal biogenesis with mitochondrial dysfunction in chronic kidney disease (CKD).

**Methods:**

Human renal tubular cells (HK-2) were cultured (+/- zinc, or 5mM/20mM glucose). mRNA levels were quantified using real-time qPCR. Transcriptomics data were retrieved and analysed from Nakagawa chronic kidney disease (CKD) Dataset (GSE66494) and Kidney Precision Medicine Project (KPMP) (https://atlas.kpmp.org/). Protein levels were determined by immunofluorescence and Western blotting. Cellular respiration was measured using Agilent Seahorse XF Analyzer. Data were analysed using one-way ANOVA, Students’ t-test and Pearson correlation.

**Results:**

The *NSA2* gene, on human chromosome 5q13 was next to *GFM2*. The two genes were syntenic on opposite strands and orientation in multiple species. Their common 381 bp 5’ region contained multiple transcription factor binding sites (TFBS) including the zinc-responsive transcription factor MTF1. *NSA2* and *GFM2* mRNAs showed a dose-dependent increase to zinc in-vitro and were highly expressed in proximal tubular cells in renal biopsies. CKD patients showed higher renal *NSA2/GFM2* expression. In HK-2 cells, hyperglycaemia led to increased expression of both genes. The total cellular protein content remained unchanged, but GFM2 upregulation resulted in increased levels of several mitochondrial oxidative phosphorylation (OXPHOS) subunits. Furthermore, increased GFM2 expression, via transient transfection or hyperglycemia, correlated with decrease cellular respiration.

**Conclusion:**

The highly conserved synteny of *NSA2* and *GFM2*, their shared 5’ region, and co-expression in-vitro and in CKD, shows they are co-regulated. Increased GFM2 affects mitochondrial function with a disconnect between an increase in certain mitochondrial respiratory proteins but a decrease in cellular respiration. These data link the regulation of 2 highly conserved genes, *NSA2* and *GFM2*, connected to ribosomes in two different cellular compartments, cytosol and mitochondria, to kidney disease and shows that their dysregulation may be involved in mitochondrial dysfunction.

**Supplementary Information:**

The online version contains supplementary material available at 10.1186/s10020-024-00930-8.

## Background

Diabetic nephropathy, the leading cause of chronic kidney disease (CKD), affects more than 800 million individuals worldwide, comprising of > 10% of the world’s population (Kovesdy 2022). CKD is a long-term condition that progresses slowly, is asymptomatic in its early stages, and despite treatment, a significant proportion of patients progress to end-stage kidney disease (Kovesdy 2022; Gaitonde et al. 2017). Diabetic nephropathy is characterised by glomerulosclerosis and tubulointerstitial fibrosis. Tubulointerstitial fibrosis affects renal tubular cells (Alsaad and Herzenberg 2007). TGF-β1 is a major mediator of fibrosis. In patients with diabetic nephropathy, TGF-β1 can mediate epithelial-to-mesenchymal transition (EMT), causing tubular epithelial cells to lose epithelial characteristics and develop myofibroblast characteristics, resulting in the development of fibrosis (Efstratiadis et al. 2009; Hills and Squires 2010). By understanding the molecular mechanisms that damage the glomerulus and the tubular cells in the kidney, novel strategies for therapeutic intervention and prevention of CKD may be identified.

We previously reported circulating *Nop-7-associated2 (NSA2)* as a biomarker of diabetic nephropathy whose elevated expression was associated with proteinuria independently of glomerular filtration rate (Shahni et al. 2012). *NSA2* was cloned in a screen for glucose regulated genes from diabetic rat kidneys, was highly conserved, and abundantly expressed in renal glomerular and tubular cells (Shahni et al. 2012). In experimental models, NSA2 mRNA/protein were increased in hyperglycaemic conditions in human renal glomerular mesangial cells (HMCs) and in kidneys and blood from diabetic mouse models (Shahni et al. 2012). NSA2 expression was linked to TGF-β1 pathway exogenous TGF-β1 increased NSA2 mRNA/protein expression accompanied by translocation of cytosolic NSA2 protein to the nuclear compartment in HMCs and in human embryo kidney cell line (HEK293), and NSA2 knock down resulted in blocking of TGF-β1 induced pathway (Shahni et al. 2013). These data strongly suggested that altered expression of NSA2 may play a mechanistic role in the development of diabetic nephropathy.

The cellular function of the NSA2 protein has been linked to protein synthesis, specifically ribosome biogenesis (Lebreton et al. 2006). Ribosomes are abundant cellular structures comprised of the large (60S) and the small (40S) subunits, each of which comprises of multiple subunits which together function as the site of protein synthesis, a high energy-requiring process, to translate mRNA into peptides. Yeast NSA2, which shows 100% identity to human NSA2, is essential for ribosomal 60S subunit assembly and biogenesis and is needed for the maturation of the peptidyl transfer centre (Lebreton et al. 2006; Paternoga et al. 2020). Data suggest that yeast NSA2 serves as a ribosome biogenesis factor and shuttles between the cytosol and nucleus during ribosomal biogenesis (Paternoga et al. 2020). To understand the potential mechanisms by which hyperglycaemia could regulate renal NSA2 expression and contribute to the mechanistic changes that lead to CKD, we evaluated potential genetic regulators of the human *NSA2* gene in the current study. Whilst examining the 5’ non-coding region of the functional human *NSA2* gene in the human genome, we found a gene known as the *GTPase-dependent ribosomal recycling factor mitochondrial 2 (GFM2)* located on the opposite strand, with the two genes sharing a common 5’ regulatory region. GFM2 is involved in mitochondrial protein synthesis, mediating the disassociation of mitochondrial ribosomal subunits from mitochondrial mRNA (Hammarsund et al. 2001; Tsuboi et al. 2009). Mutations in *GFM2* can cause impaired protein synthesis within the mitochondrial respiration chain and have been found to be associated with the onset of Leigh syndrome and subsequent impairment of mitochondrial oxidative phosphorylation (Fukumura et al. 2015; Glasgow et al. 2017).

Translation of mRNA to protein takes place in both the cytoplasm and mitochondria in eukaryotic cells (Boczonadi & Horvath 2014). Mitochondria possess an independent protein synthesis system comprising of a large (39 S) and small (28 S) ribosome subunit, which resemble prokaryotic ribosomes, and specific tRNAs which use a slightly different genetic code to translate mtDNA encoded mRNAs into proteins (Richter-Dennerlein et al. 2016; Wang et al. 2021).

The synteny of *GFM2* to *NSA2* genes was highly intriguing since both seem to be involved in ribosome functions and hence protein synthesis and shared what seemed to be a common 5’ non-coding region in the genome. Therefore, we postulated that the two genes may share common transcriptional regulators, and furthermore, we proposed that both may be aberrantly expressed in CKD possibly contributing to ribosomal and mitochondrial dysfunction. This paper describes studies of the 5’regulatory region of the 2 genes *NSA2* and *GFM2*, the increased expression and correlation of the expression of the two genes in human renal biopsy tissue from patients with CKD, and the increased protein content of mitochondrial respiration chain as well as altered mitochondrial respiration in hyperglycaemic renal cells with elevated GFM2 expression. Together, these data provide evidence that ribosome function and, hence, protein translation in the cytosol and mitochondria is co-regulated in cells and is altered in CKD, which may contribute to mitochondrial dysfunction. Evaluating the expression of GFM2 and mitochondrial respiration in diabetic renal cells may help understand the role of ribosome function in the mechanism of diabetic nephropathy.

## Methods

### HK-2 cell culture

The human proximal renal tubular cell line, HK-2, was cultured in Dulbecco’s Modified Eagle Medium (DMEM, #22320022, Thermo Fisher, UK) with 10% fetal bovine serum (#F0804, Sigma, UK) and 1% penicillin-streptomycin (#15240062, Thermo Fisher, UK) at 37℃ in 95% air and 5% CO_2_. The passage number was below 30. Media was changed every 2 days. Cells were arrested at around 80% confluency and seeded in triplicate at a density of 2 × 10^5^ cells, as previously described (Czajka and Malik 2016). To investigate the impact of the zinc-sensing metal-responsive transcription factor 1 (MTF1) on gene expression, HK-2 cells were seeded in triplicate in 6-well plates and synchronized by growth in serum-free media for 1 h, followed by growth in DMEM without or with additional zinc sulphate at the concentration of 20µM or 40µM for 24 h (Hogstrand et al. 2008). For the glucose regulation experiments, cells were treated in DMEM containing 10% fetal bovine serum with normal glucose (NG = 5mM glucose) or high glucose (HG = 20mM glucose) for 2, 4, 6 and 8 days. Cell pellets were collected and stored at -80℃ for RNA or protein extraction.

### RNA extraction and real-time quantitative PCR

Total RNA was extracted from HK-2 cell pellets using RNeasy Mini Kit (#74106, Qiagen, UK), treated with DNase-I to remove DNA contamination (#AMPD1, Sigma, UK), and reverse transcribed to cDNA using random primers, oligonucleotides dNTP (#10297018, ThermoFisher, UK) and reverse transcriptase (#M170A, Promega, UK). Oligonucleotide primers were designed using NCBI Blast-primer and were synthesized from Integrated DNA Technologies Company (Table 1). Real-time quantification PCR (qPCR) was carried out using SYBR green (#204057, Qiagen, UK). The qPCR reaction was conducted as 5-min pre-incubation at 95℃, 40 cycles of denaturation (10s, 95℃) and annealing/extension (30s, 60℃), melting at 95℃ for 5s and 65℃ for 60s, and cooling at 4℃ for 30s using LightCycler 96 (Roche, Germany). The absolute copy number of *NSA2*, *GFM2* and *GAPDH* mRNA is based on the standard curve method (Shahni et al. 2012). *NSA2* and *GFM2* mRNA content was calculated by normalising its copy number to the reference gene *GAPDH* mRNA copy number.

**Table 1:** Oligonucleotide primers used in this study

Accession no.	Name	Oligonucleotide sequence	Product size(bp)
NM_032380	hGFM2 F2	AGTTCTCCATGACAAGCAGCG	143
	hGFM2 R2	GTTGGTCAGCAAACGGCAA	
NM 014886	hNSA2 F6	AGAAGGCGGGAAAATGGGAAG​	253
	hNSA2 R6	ATTGGTAGGCAAAAGGTGGCT	
NM_002046	hGAPDH F1	TGCACCACCAACTGCTTAGC	87
	hGAPDH R1	GGCATGGACTGTGGTCATGAG	

### Immunofluorescent staining

HK-2 cells were grown on coverslips and fixed with 4% paraformaldehyde. The fixed cells were incubated with chicken anti-NSA2 primary antibody (#ABIN478277, Antibodies-online) at 4℃ overnight and goat anti-chicken Alexa Fluor 488 conjugated secondary antibody (#ab150173, Abcam) for 1 h to stain NSA2, and incubated with rabbit anti-GFM2 primary antibody (#PA5-85482, Invitrogen) overnight and goat anti-rabbit Cyanine5 conjugated secondary antibody (#A10523, Invitrogen) for 1 h to stain GFM2. the cells were mounted on the slides using DAPI Fluoromount-G (#0100 − 20, Southern Biotech), which stained the nucleus in blue. The slides were kept in the dark until dry and examined under Nikon A1-inverted fluorescence microscope. The fluorescence integrated density was measured using ImageJ software.

### Transient transfection

The full-length human *GFM2* cDNA was fused with a V5 tag (GKPIPNPLLGLDST) and was cloned into the expression vector pRP(Exp) by VectorBuilder Company. The intended construct was confirmed by restriction enzyme digestion and Sanger sequencing.

Before transfection, HK-2 cells were cultured in 6-well plates or 96-well plates until reaching 70 − 90% confluent. The transient transfection was conducted using Lipofectamine™ 3000 transfection reagent (#L3000008, Thermo Fisher Scientific, UK) following the manufacturer’s protocol. Initially, Lipofectamine 3000 was diluted in Opti-MEMTM medium (#31985062, Thermo Fisher Scientific, UK). Simultaneously, plasmid DNA was mixed with P3000 and Opti-MEM™ medium. The diluted DNA was then mixed with diluted Lipofectamine 3000 at the ratio of 1:1 and incubated at room temperature for 10–15 min to form plasmid DNA-lipid complexes (lipoplex). The resultant mixture was added into each well containing cells and growth media. The cells were then cultured for 24 h at 37 °C and subjected to RNA, protein, or mitochondrial respiration analysis.

### Western blotting

Collected cell pellets were lysed in ice- cold RIPA lysis buffer (#R0278, Sigma-Aldrich, UK) containing PierceTM Protease Inhibitor (#A32955, Thermo Fisher Scientific, UK) and PhosStop phosphatase inhibitors (#04906845001, Roche, Germany) at the ratio of 100µL lysis buffer per 10^6^ cells. The total protein content was quantified using using Pierce™ BCA Protein Assay Kit (#23225, Thermo Fisher Scientific, UK).

Protein solutions were prepared at the concentration of 2 µg/µL mixed with NuPAGE™ LDS Sample buffer (4X), NuPAGE™ Sample reducing agent (10X) and deionised water. 40 µg of solubilized protein extract was loaded on NuPAGE 4–12% Bis-Tris Gel (#NP0322BOX, Invitrogen), electrophoresed at 100 V for 30 min, followed by 150 V for 1 h. PageRuler™ Plus Prestained Protein Ladder (#26619, Thermo Fisher Scientific, UK) was loaded to help evaluate the size of the targeted proteins. Subsequently, the proteins on the gel were transferred to a PVDF membrane activated by methanol. Immunodetection was performed using Rabbit anti-GFM2 primary antibody (#PA5-85482, Invitrogen), HRP Anti-V5 tag antibody (#ab173837, Abcam), Total OXPHOS Rodent WB Antibody Cocktail (#ab110413, Abcam), IRDye^®^ 680RD Goat anti-Mouse IgG Secondary Antibody (#P/N 926-68070, Licor), and IRDye^®^ 800CW Goat anti-Rabbit IgG Secondary Antibody (#P/N 926-32211, Licor). The total protein of each lane was stained using Licor Revert 700 Total Protein Stain Kit (Li-Cor Biosciences #926-11011, UK). The signal was quantified using Licor Image Studio™ software. The normalisation factor was determined by calculating the ratio of the total protein signal for each lane to the signal from the lane with the loading control (Eq. [1]). The normalised signal of target proteins was calculated as the ratio of the target band signal to the lane normalisation factor (Eq. [2]). In this study, the same protein solution extracted from HEK293 cells was loaded on each Bis-Tris Gel as the loading control.

1 $$\eqalign{& lane\,normalisation\,factor = \cr & {{total\,protein\,signal\,for\,each\,lane} \over {total\,protein\,signal\,from\,the\,lane\,with\,the\,loading\,control}} \cr}$$

2 $$normalised\:signal=\frac{target\:band\:signal}{\:lane\:normalisation\:factor}$$

### Mitochondrial respiration analysis

Mitochondrial respiration of HK-2 cells was assessed using Seahorse analyser and XF Cell Mito Stress Test Kit (#103015-100, Aligent Technologies, USA). One day before the assay, HK-2 cells were seeded in 96-well Seahorse cell plates at the density of 2 × 10^4^ cells per well. The Seahorse analyser measured oxygen consumption rate (OCR) as an indicator of mitochondrial respiration.

Throughout the assay, 1.5µM Oligomycin, 0.5µM Carbonyl cyanide-4 (trifluoromethoxy) phenylhydrazone (FCCP), and 0.5µM Rotenone and Antimycin A were sequentially injected. In mitochondrial electron transport chain (ETC), ATP synthase can catalyse ADP to ATP. The injection of Oligomycin can inhibit ATP synthase, thereby blocking ATP-linked respiration. The addition of FCCP, a potent uncoupler, can disrupt mitochondrial membrane potential and collapse the proton gradient. This, in turn, prompts unrestricted electron flow through the ETC, allowing the OCR to achieve its maximum level. The range between maximum and basal respiration levels reflects mitochondrial spare capacity. The spare capacity becomes active under stress conditions when energy demand increases with more ATP required to maintain cellular functions. After the injection of rotenone and antimycin A, the inhibitor of Complex I and III, the mitochondrial respiration was completely shut down, and the OCR level corresponds to non-mitochondrial respiration. After the OCR measurements, the cells were lysed with 10µL of RIPA buffer per well and diluted in deionised water as 1:5 for BCA assay to quantify protein content. The OCR levels per well were normalised to protein content and presented as pmolesO_2_/min/µg protein.

### Bioinformatics

DNA sequence analysis and conservation assessment were conducted to investigate the potential common and conserved transcriptional factor binding sites (TFBS) of *NSA2* and *GFM2* genes. Potential TFBS were identified by reviewing the sequences within the shared 5’ regulatory region of both genes using Multitf ( https://multitf.dcode.org/). To assess the conservation of the potential common transcription factors, the 5’ overlapping sequence was aligned across multiple species. The genetic arrangement of *NSA2* and *GFM2* genes in various species was reviewed using the NCBI GeneBank (https://www.ncbi.nlm.nih.gov/genbank/). The organization and orientation of these genes were examined across different species to evaluate the conservation of their genetic arrangement.

### Patient dataset

The gene expression profiles of human renal biopsies were obtained from the Nakagawa CKD Kidney dataset (GSE66494) available in NCBI Gene Expression Omnibus (GEO) and Nephroseq database (http://www.ncbi.nlm.nih.gov/geo/query/acc.cgi?acc=GSE66494). The Nakagawa CKD Kidney dataset was generated using the Agilent-014850 Whole Human Genome Microarray 4 × 44 K platform (GPL6480), consisting of kidney biopsy samples from 8 healthy control (HC) patients and 45 patients with CKD (Nakagawa et al. 2015). The Kidney Precision Medicine Project (KPMP) kidney tissue atlas (https://atlas.kpmp.org/explorer/dataviz) was used to access the single-cell RNA-seq dataset derived from renal biopsies from 20 HC and 15 CKD patients (Hansen et al. 2022). *NSA2* and *GFM2* mRNA levels were evaluated in 16 main renal cell clusters comprised of 57 subtypes.

### Statistics

The gene expression level in multiple groups was compared using one-way ANOVA test and Tukey’s test. Student’s t-test was used for comparisons between two groups. The data of each group were shown in bar charts as mean ± standard deviation, and *p* < 0.05 suggested the significant difference between groups. Pearson coefficient (r) was used to determine the correlation of *NSA2* and *GFM2* gene expression levels. r level varies between − 1 and 1, where 0 represents no correlation, 1 represents total positive correlation, and − 1 represents total negative correlation. *p* < 0.05 suggested the correlation was statistically significant. All the statistical analyses were performed using GraphPad Prism 9 software (GraphPad Software, San Diego, CA).

## Results

### The 5’ region of the human *NSA2* gene and the synteny of *NSA2* and *GFM2*

**Fig. 1:**
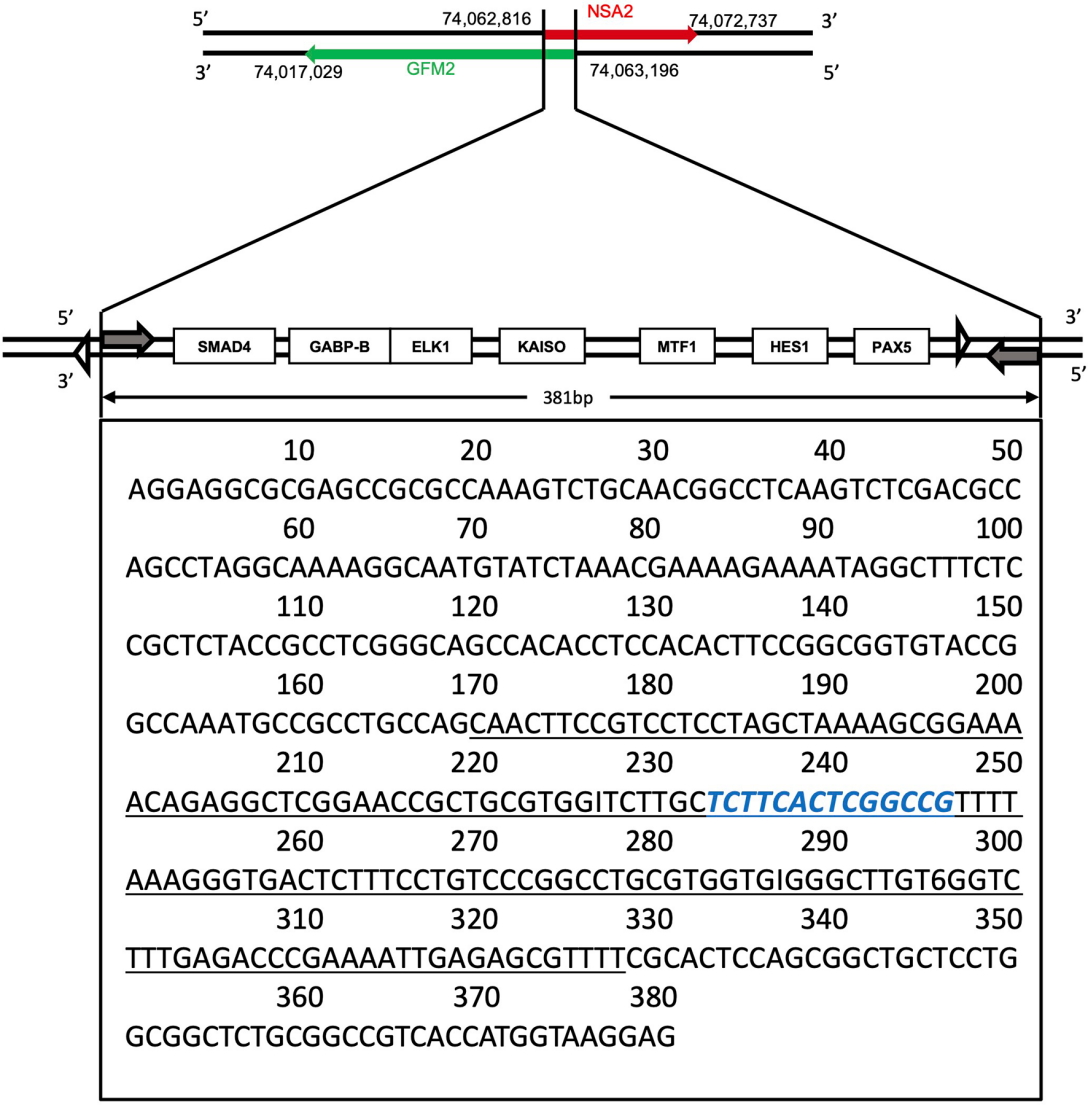
The 5’ region of *NSA2* gene on human chromosome 5. The black double strands represent human chromosome 5, with the red arrow denoting the transcriptional directions of *NSA2*. On the opposite, *the* green arrow denotes the transcriptional directions of *GFM2* at the 5’ side of *NSA2*. Numeric labels accompanying the double strands provide the exact locations of these genes on human chromosome 5. 381 bp 5’ overlapping region is magnified. NSA2 transcription initiation point (shaded arrow) and translation start codon (white triangle) are labelled on the upper strand, while those of GFM2 are shown on the bottom strand. Transcription factor binding sites (TFBS) distribution within the overlapping region are ordered based on their positions. The full-length conserved overlapping sequences between human and *Macaca mulatta* (monkey) are shown in the black box. The conserved region in humans, monkeys and mice is underlined. The conserved MTF1 binding site is in Blue. SMAD4, Mothers against decapentaplegic homolog 4; GABP-B, GA Binding Protein Transcription Factor Subunit Beta; ELK1, ETS Like-1; KAISO, Zinc Finger and BTB Domain Containing 33; MTF1, Metal Regulatory Transcription Factor 1; HES1, Hes Family BHLH Transcription Factor 1; PAX5, Paired Box Gene 5

The functional human *NSA2* gene sequence is located on human chromosome 5q13 at 74,062,816–74,072,737 in human genome Assembly hg19 (Fig. 1). Seven TFBS were identified within 400 bp upstream of the *NSA2* coding region. The exact 5’ region position of each transcription factor and their potential relationship to NSA2 regulation is given in Additional file 1. We found that the gene *GFM2*, located at Chromosome 5: 74,017,029–74,063,196, was on the complementary strand to *NSA2* and situated adjacent to *NSA2*, with the two genes sharing a common 381 bp 5’ non-coding regulatory region containing several TFBS (Fig. 1).

**Fig. 2:**
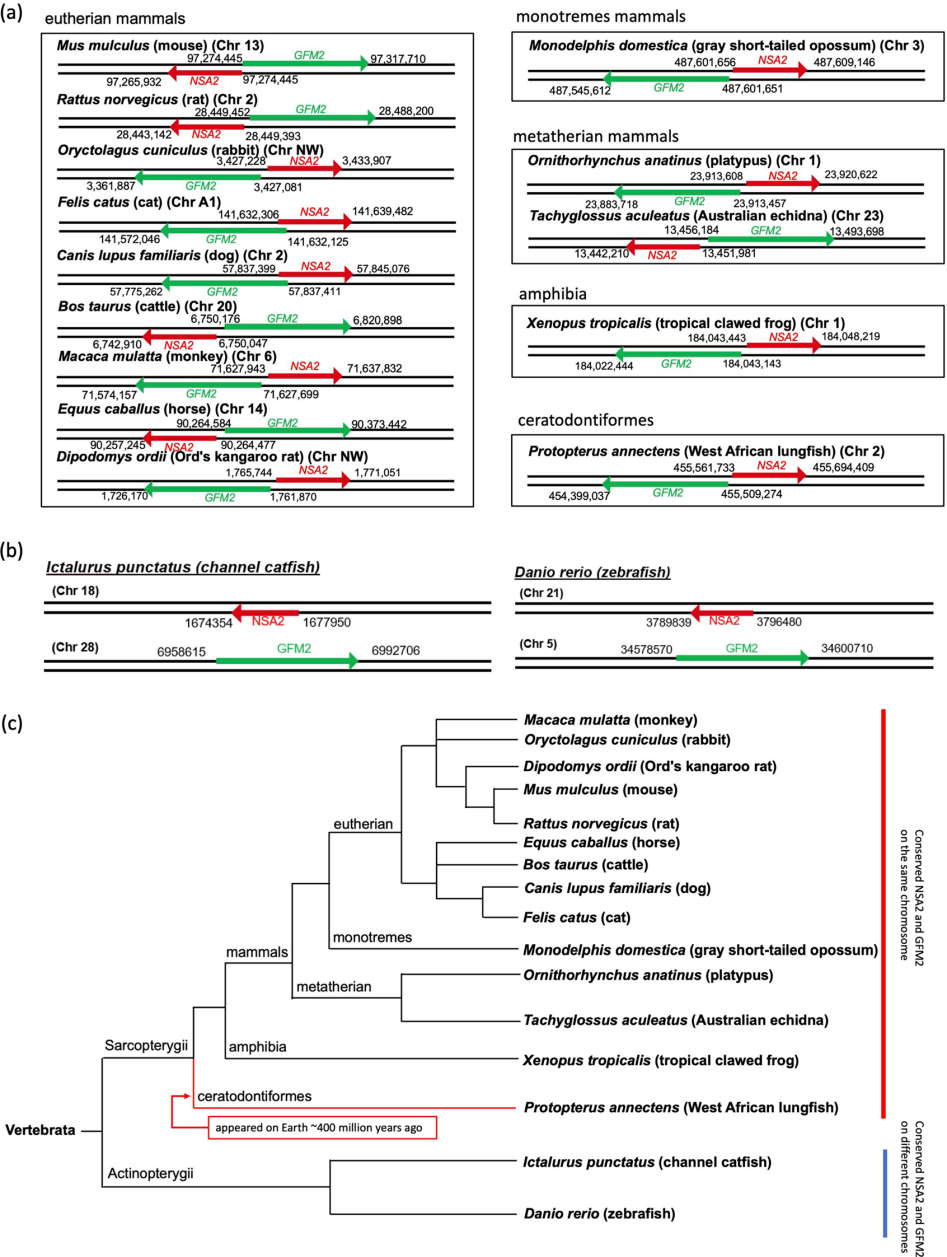
The synteny of *NSA2* and *GFM2* in vertebrata species. The black lines in (**a**) and (**b**) represent sections of chromosomes that *NSA2* and *GFM2* are on, and the arrow shows the transcription direction from 5’ to 3’. The names of the species are labelled at the left top of each chromosome. The numbers represent the specific location of the genes. *NSA2* and *GFM2 are* syntenic in multiple species (**a**) but not in catfish and zebrafish (**b**). The classification of the species is labelled on top of the square. The evolution tree (**c**) of the species involved in (**a**) and (**b**) was generated using Lifemap NCBI version. Ceratodontiformes lungfish appeared on Earth around 400 million years ago (Jorgensen and Joss 2010). Chr, chromosome

The gene arrangement of human *NSA2* and *GFM2* was evaluated in eukaryotic species. As in humans, the *NSA2* and *GFM2* genes are located on the same chromosome, on complementary strands and opposite orientation, and share a common 5’ regulatory region in multiple eukaryotic species (Fig. 2a). This synteny is conserved in eutherian mammals (mouse, rat, rabbit, cat, dog, cattle, horse, monkey, and kangaroo), monotremes mammals (platypus and Australian echidna), metatherian mammals (gray short-tailed opossum) and non-mammals, like amphibia (frog), and ceratodontiformes (lungfish) (Fig. 2a). However, the synteny of *NSA2* and *GFM2* is not conserved in catfish and zebrafish, where the two genes are located on two different chromosomes (Fig. 2b). The species catfish and zebrafish, where there is no synteny seen between *GFM2* and *NSA2* (Fig. 2b), are part of the Actinopterygii branch which diverged from the Sarcopterygii branch of vertebrata (Fig. 2c). Sarcopterygii, comprises ceratodontiformes, amphibia and mammals (Fig. 2c), in which we show the synteny of *NSA2* and *GFM2* (Fig. 2a).

Although *NSA2* and *GFM2* are syntenic in multiple species, the non-coding 381 bp overlapping sequence, including TFBS, is not evolutionarily conserved. Sequence alignment showed that the entire 381 bp region was only conserved in *Macaca mulatta* (monkey) with 92.4% identity, whereas 159 bp was conserved in *Mus musculus* (mouse) with 68.6% identity (Table 2; Fig. 1). One common evolutionary conserved TFBS between monkeys, mice and human beings is MTF1, a metal-responsive transcription factor (Fig. 1). In human proximal renal tubular cell line (HK-2) grown in the presence and absence of added zinc, the transcription of *NSA2* and *GFM2* mRNAs showed a dose-dependent increase upon the addition of zinc (Additional file2). These results show that zinc can control the expression of both *NSA2* and *GFM2 in vitro*, probably by activating MTF1.

**Table 2:** Evolutionary conserved regions from human *NSA2*-*GFM2* overlapping region

Species	length	Percent identity	Relative position*	Human genomic position
Macaca mulatta	381 bp	92.4%	1-381	chr5:74062816–74,063,196
Mus musculus	159 bp	68.6%	170–328	chr5:74062985–74,063,143

*Relative position is the position in human *NSA2*-*GFM2* overlapping region

### Co-expression of renal *NSA2* and *GFM2* in patients with chronic kidney disease

**Fig. 3:**
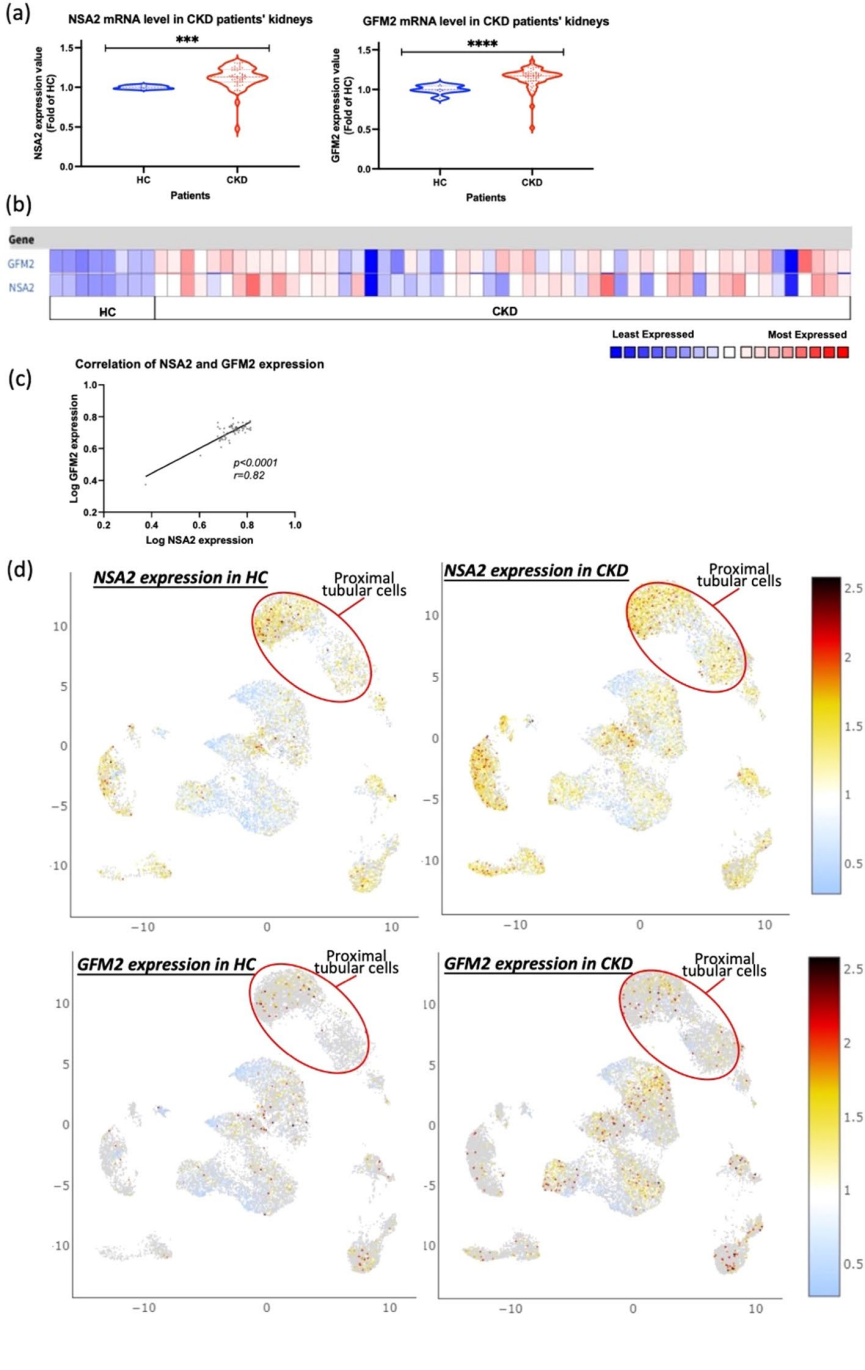
Co-regulation of *NSA2* and *GFM2* mRNA expression in patients with chronic kidney disease. (**a**) The mRNA expression value and correlation of *NSA2* and *GFM2* in human kidney biopsies between chronic kidney disease (CKD) and healthy control (HC) patients. The mRNA expression values of both genes were obtained from the Nakagawa chronic kidney disease (CKD) Kidney Dataset. The expression level is shown as fold change to HC. (**b**) The heat map of *NSA2* and *GFM2* in human kidney biopsies between CKD and HC patients. The low expression levels are shown in blue, and the high expression levels are in red. (**c**) The correlation of *NSA2* and *GFM2* in human kidney biopsies. The Pearson correlation efficiency *r* = 0.82, *p* < 0.0001. HC: *n* = 8. CKD: *n* = 45. Data Links: http://www.ncbi.nlm.nih.gov/geo/query/acc.cgi?acc=GSE66494 (**d**) Differential expression of *NSA2* and *GFM2* in kidney cell clusters from HC and CKD in Kidney Precision Medicine Project (KPMP) kidney tissue atlas. HC, *n* = 20. CKD, *n* = 15. Data link: https://atlas.kpmp.org/explorer/dataviz

As both *NSA2* and *GFM2* share the common 5’ regulatory region, suggesting co-regulation, we evaluated the mRNA expression levels of both genes in renal biopsies from HC and CKD patients. Using the Nakagawa CKD Kidney Dataset (Nakagawa et al. 2015), which comprises of microarray gene expression profiles from 53 biopsy specimens of CKD and HC patients, the expression values of both *NSA2* and *GFM2* were found to be significantly higher in CKD group than in the HC group (*NSA2 p* < 0.001, *GFM2 p* < 0.0001, Fig. 3a), and the regulation trend of *NSA2* and *GFM2* were similar as indicated by the heatmap (Fig. 3b). Pearson’s correlation analysis indicated that renal mRNA levels of *NSA2* and *GFM2* were significantly correlated to one another in human kidney (*p* < 0.0001, *r* = 0.82, Fig. 3c).

In the single-cell RNA-seq dataset from the KPMP kidney tissue atlas, *NSA2* and *GFM2* mRNA levels were evaluated (Hansen et al. 2022). The reference UMAP comprised of 16 main renal cell clusters containing 57 subtypes from which we located the proximal tubular cell cluster to evaluate expression levels of *NSA2* and *GFM2* in HC and CKD biopsies (Additional file 3 and 4). *NSA2* expression was relatively high in the proximal tubular cell cluster compared to that in the other renal cell clusters in HC (Fig. 3d). Similarly, proximal tubular cell cluster exhibited a relatively high level of *GFM2* expression in comparison to the majority of other clusters in HC patients (Fig. 3d). In CKD patients, a higher expression of *NSA2* and *GFM2* was observed compared to HC (Fig. 3d).

### Glucose-induced upregulation of NSA2 and GFM2 in parallel with altered mitochondrial OXPHOS subunits and respiration in *vitro*

#### High glucose increases mRNA and protein levels of both NSA2 and GFM2 in renal tubular cells

**Fig. 4:**
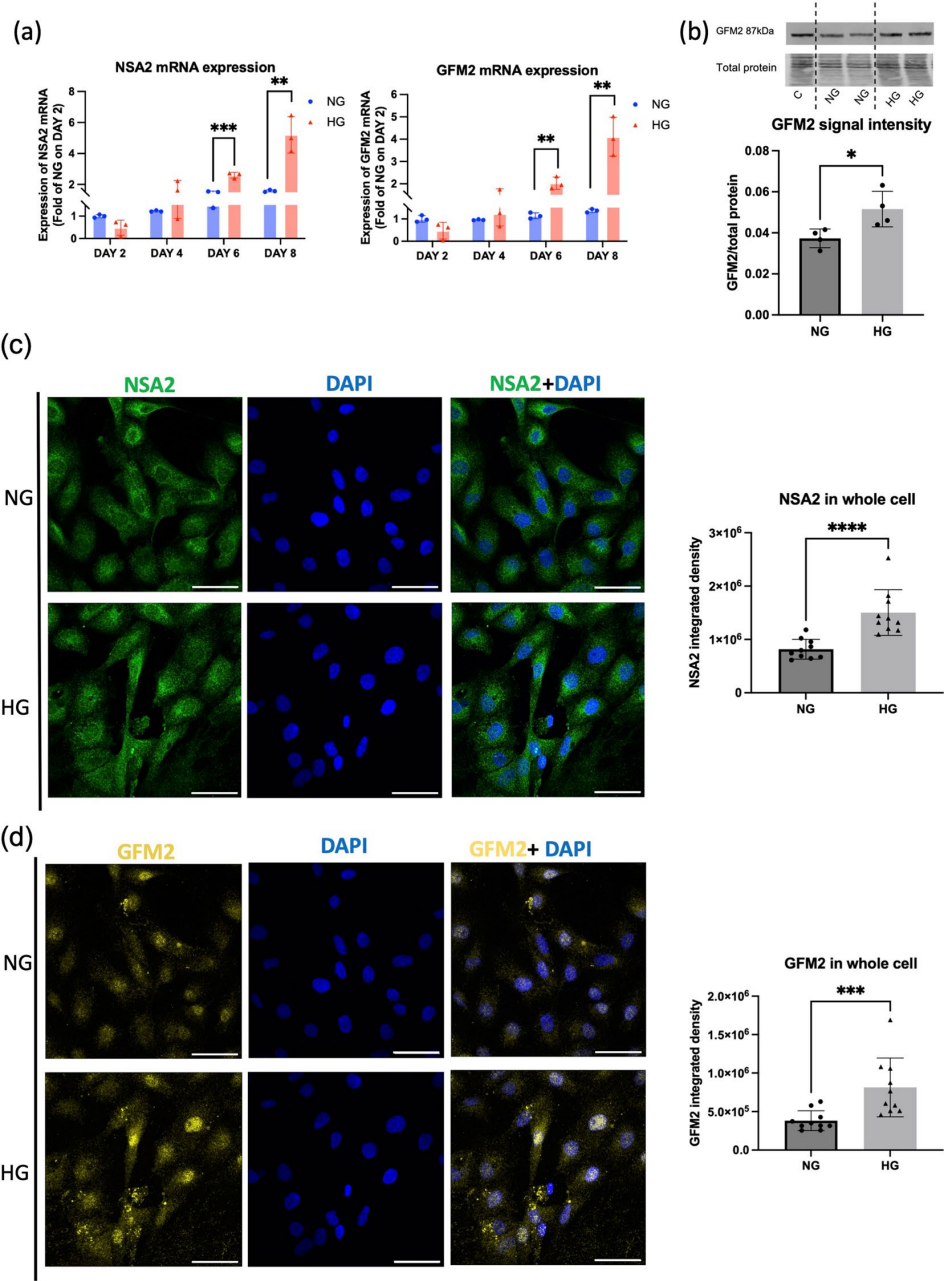
Upregulation of NSA2 and GFM2 in HK-2 cells exposed to high glucose. *NSA2* and *GFM2* mRNA expression level fold changes in HK-2 exposed to normal (NG = 5mM glucose) or high glucose (HG = 20mM glucose). (**a**) The *NSA2* and *GFM2* mRNA expression levels were calculated as mRNA copy numbers relative to per 1000 *GAPDH.* The fold changes were normalized to the mRNA expression level in NG on Day 2. *N* = 3. (**b**) Western blot of GFM2 in HK-2 cells grown in NG and HG for 6 days. The same loading control (C) prepared from HEK293 cells was used in all westerns. The normalised signal of GFM2 protein was calculated as the ratio of the target band signal to the lane normalisation factor. Immunofluorescent staining of NSA2 (**c**) and GFM2 (**d**) in HK-2 cells exposed to NG and HG for 6 days. NSA2 protein was labelled using chicken anti-NSA2 primary antibody and goat anti-chicken Alexa Fluor^®^ 488 secondary antibody (green). GFM2 protein was labelled using rabbit anti-GFM2 primary antibody and goat anti-rabbit Cyanine5 secondary antibody (yellow). Nucleus was stained using DAPI (blue). The scale bar represents 50 μm. The data were analysed using Students’ t-test. * p < 0.05, ** p < 0.01, *** p < 0.001, **** p < 0.0001.N = 10 for each group

We previously showed that *NSA2* mRNA is upregulated in high glucose in renal mesangial cells, however, its expression or glucose regulation in human renal tubular cells has not been demonstrated. To evaluate the potential co-expression of *NSA2* and *GFM2*, we evaluated whether *NSA2* is regulated by glucose in human tubular cells and whether GFM2 shows similar expression patterns to NSA2 at mRNA and protein levels. In HK-2 exposed to NG or HG media for 6 days, the mean *NSA2* and *GFM2* mRNA copy number relative to *GAPDH* gene in NG were 0.011 and 0.253, respectively, while the mRNA content of *NSA2* and *GFM2* was significantly increased to ~ 2 fold in HG (*n* = 3, *NSA2* = 0.022 *p* < 0.001, *GFM2* = 0.045 *p* < 0.01) (Fig. 4a). HG-induced upregulation of *NSA2* and *GFM2* mRNA was also statistically significant after 8 days’ HG exposure (*n* = 3, *NSA2 p* < 0.001, *GFM2 p* < 0.01). In HK-2 cells cultured in NG and HG for 6 days, Western blotting results showed significant increase of GFM2 signal in HG compared to in NG (Fig. 4b). Changes of NSA2 and GFM2 protein levels in response to high glucose were evaluated by immunofluorescent staining. In HK-2 cells, NSA2 and GFM2 fluorescence integrated density in HG was significantly higher than in NG (Fig. 4c-d). These results indicate that NSA2 and GFM2 are up-regulated by high glucose at mRNA and protein levels in human proximal renal tubular cells in vitro (Fig. 4).

#### High glucose induced NSA2 and GFM2 up-regulation is parallel to increased levels of mitochondrial OXPHOS subunits in tubular cells

**Fig. 5:**
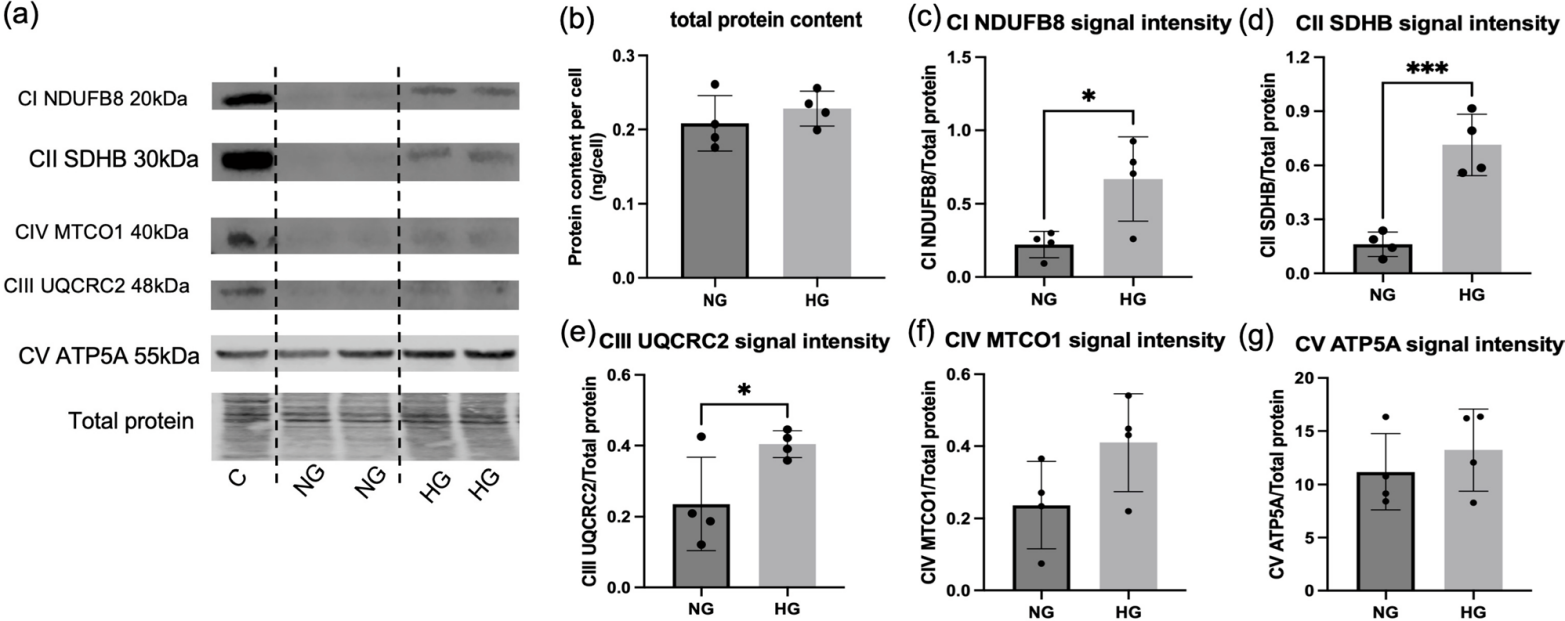
Mitochondrial OXPHOS and cellular total protein content changes in HK-2 cells exposed to high glucose. (**a**) Western blot of 5 mitochondrial OXPHOS proteins in HK-2 cells grown in normal (NG = 5mM) and high glucose (HG = 20mM) for 6 days. Proteins in the loading control (C) were from HEK293 cells. (**b**) Total protein content in HK-2 cells in NG and HG. The total protein content was measured using Bicinchoninic acid assay. (**c**-**g**) Protein signal intensity of 5 mitochondrial OXPHOS proteins tested by Western blotting in HK-2 cells. The normalisation factor was determined as the ratio of the total protein signal for each lane to the signal from the loading control. The normalised signal of target proteins was calculated as the ratio of the target band signal to the lane normalisation factor. The data were analysed using Students’ t-test. * *p* < 0.05, ** p < 0.01, *** p < 0.001.N = 4. CI, Complex I; CII, Complex II; CIII, Complex III; CIV, Complex IV; CV, Complex V; NDUFB8, NADH dehydrogenase [ubiquinone] 1 beta subcomplex subunit 8; SDHB, Succinate Dehydrogenase Complex, Subunit B; MTCO1, Mitochondrially Encoded Cytochrome C Oxidase I; UQCRC2, Ubiquinol-Cytochrome C Reductase Core Protein 2; ATP5A, ATP Synthase F1 Subunit Alpha

Since NSA2 and GFM2 are associated with ribosomal functions in cytosol and mitochondria, respectively, we evaluated whether their upregulation in high glucose coincides with increased cellular and/or mitochondrial protein levels. The total protein content per cell, at 0.21 ± 0.037 ng to 0.23 ± 0.024 ng, in NG and HG respectively, did not show an increase (Fig. 5b). However, selected subunits of the mitochondrial OXPHOS system, including nuclear-genome encoded and mitochondrial-genome encoded proteins, were increased in parallel with NSA2 and GFM2 increase in HG (Fig. 5a). The mitochondrial OXPHOS subunits valuated were NDUFB8 (Complex I), SDHB (Complex II), UQCRC2 (Complex III), and ATP5A (Complex V) encoded by the nuclear genome, and MTCO1 (Complex IV) encoded by the mitochondrial genome. HG significantly increased the protein level of NDUFB8, SDHB, and UQCRC2 in HK-2 cells (Fig. 5a, c-e). The protein levels of MTCO1 in Complex IV and ATP5A in Complex V showed a trend of increase in HG compared to NG control, but the difference was insignificant (Fig. 5f-g).

#### High glucose leads to reduction in mitochondrial respiration capacity

**Fig. 6:**
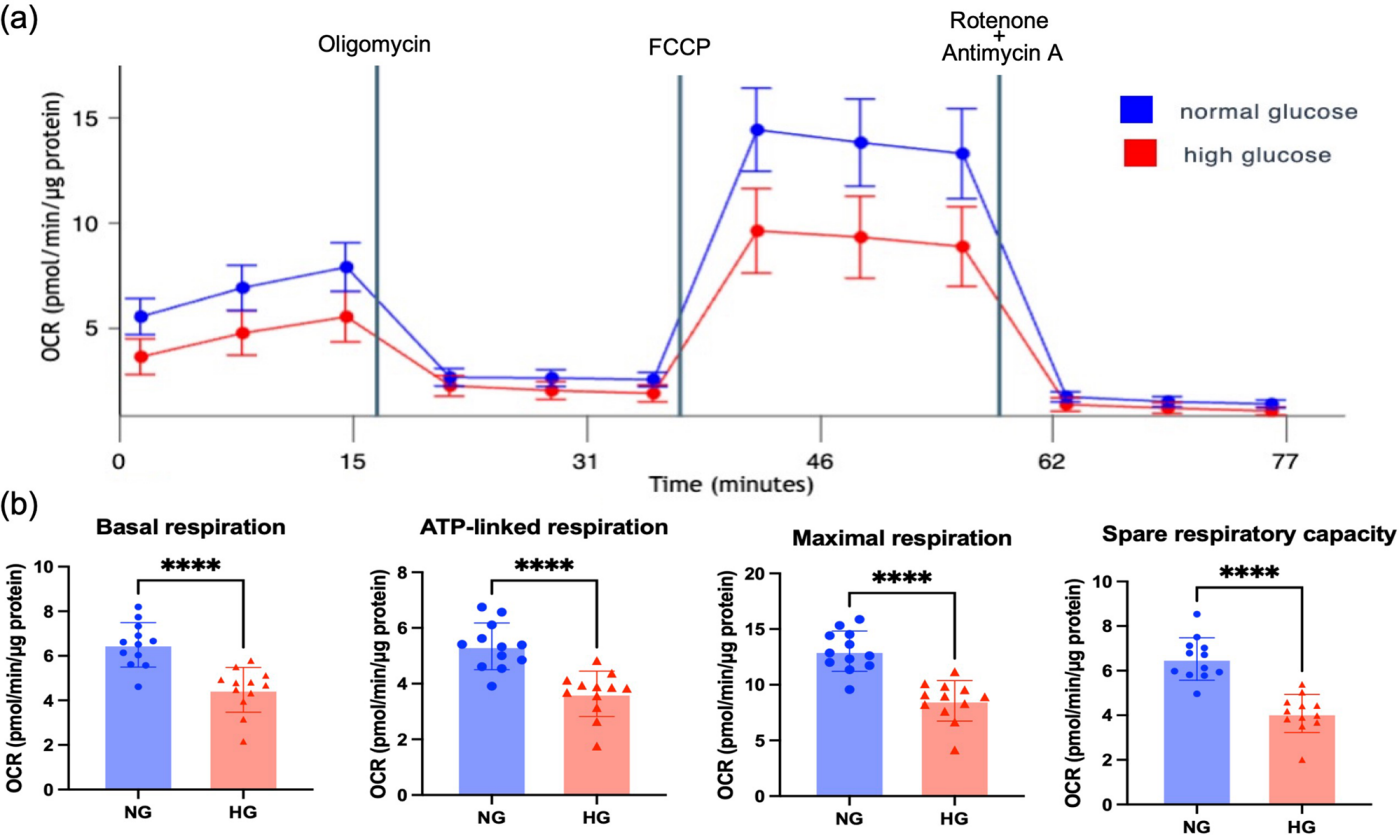
Mitochondrial respiration changes in HK-2 cells exposed to high glucose. (**a**) Respiration profile of HK-2 cells cultured in normal glucose (NG = 5mM) or high glucose (HG = 20mM) media for 6 days. Cell oxygen respiration rate (OCR) was measured using Seahorse Analyser. After the measurement of basal respiration, the injection of Oligomycin can inhibit ATP synthase, thereby blocking ATP-linked respiration. FCCP can disrupt mitochondrial membrane potential and collapse the proton gradient, allowing the OCR to achieve its maximum level. The range between maximum and basal respiration levels reflects mitochondrial spare capacity. Rotenone and antimycin A are inhibitor of Complex I and III, which can completely shut down the mitochondrial respiration. (**b**) Changes of basal respiration, ATP-linked respiration, maximal respiration, and spare respiratory capacity in HK-2 cells cultured in NG or HG media. *N* = 12. The data were analysed using Students’ t-test. **** p < 0.0001

To determine whether the elevated mitochondrial OXPHOS subunit protein levels seen in parallel with HG-induced NSA2 and GFM2 up-regulation altered mitochondrial function, cellular respiration was evaluated using the Agilent Seahorse system. Under HG conditions, the basal respiration of HK-2 cells was significantly reduced, when compared to NG control (Fig. 6a-b, NG 6.49 ± 0.95, HG 4.47 ± 0.96, *p* < 0.0001). ATP-linked respiration level was also significantly reduced in HG (Fig. 6b, NG 5.34 ± 0.80, HG 3.64 ± 0.78, *p* < 0.0001). In addition, cell maximal respiration level (NG 13.02 ± 1.73, HG 8.56 ± 1.74) and spare respiratory capacity (NG 6.52 ± 0.91, HG 4.08 ± 0.82) were significantly lower in HG than that in NG (Fig. 6b). Taken together, in human renal tubular cells, high glucose can cause increased protein levels of NSA2, GFM2 and mitochondrial OXPHOS subunits but in parallel the cells display reduced mitochondrial respiration capacity. However, whether the loss in mitochondrial respiration capacity is a consequence of increased NSA2/GFM2 or an indirect effect of HG remained unknown.

### Impact of GFM2 upregulation on mitochondrial protein content and respiration

**Fig. 7:**
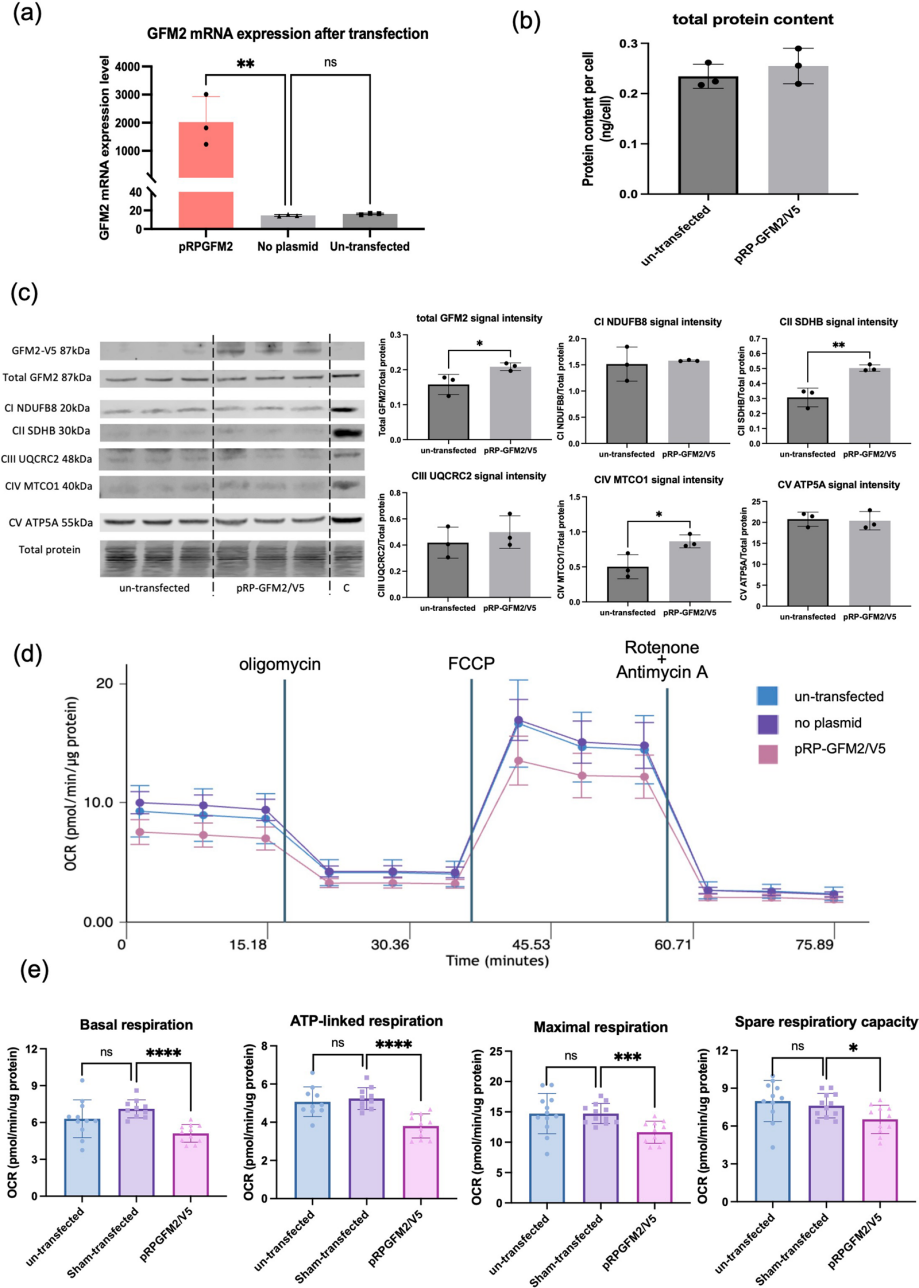
Mitochondrial OXPHOS and respiration changes in HK-2 cells with elevated GFM2 expression by transient transfection. (**a**) The mRNA expression of GFM2 in HK-2 cells after 24-hour transfection using pRP-GFM2/V5 plasmid. (**b**) Total protein content in pRP-GFM2/V5 plasmid transfected or un-transfected HK-2 cells. (**c**) Western blot of GFM2/V5 tag and OXPHOS proteins in HK-2 cells transfected with pRP-GFM2/V5 plasmid. Proteins in the loading control (C) were from HEK293 cells. The normalisation factor was determined as the ratio of the total protein signal for each lane to the signal from the loading control. The normalised signal of target proteins was calculated as the ratio of the target band signal to the lane normalisation factor. The data were analysed using Students’ t-test. * *p* < 0.05, ** p < 0.01.N = 3. (**d**) Respiration profile of un-transfected, Sham-transfected, and pRP-GFM2/V5 transfected HK-2 cells. Cell oxygen respiration rate (OCR) was measured using Seahorse Analyser as an indicator of mitochondrial respiration. (**e**) Changes of basal respiration, ATP-linked respiration, maximal respiration, and spare respiratory capacity in un-transfected, Sham-transfected, and pRP-GFM2/V5 transfected HK-2 cells. Un-transfected, HK-2 cells grown without transfection reagents and plasmids. Sham-transfected, HK-2 cells grown with transfected reagents but no plasmids. *N* = 12. The data were analysed using one-way ONOVA. *ns p > 0.05*, *p < 0.05, *** p < 0.001, **** p < 0.0001

To evaluate the impact of increased NSA2 and/or GFM2 on mitochondrial OXPHOS proteins and respiration independently of HG, we induced the increased expression of GFM2 in HK-2 cells by transient transfection with pRP-GFM2/V5 plasmid. The overexpression of GFM2 in pRP-GFM2/V5 plasmid transfected cells was confirmed by real-time qPCR and Western blotting (Fig. 7a and c). Due to the lack of NSA2 antibodies suitable for Western blotting it was not possible to assess the impact of upregulated endogenous NSA2 on mitochondrial OXPHOS proteins and respiration.

The overexpression of GFM2 achieved using pRP-GFM2/V5 plasmid did not alter cellular total protein content (Fig. 7b). In the pRP-GFM2/V5 plasmid transfected cells, the expression of GFM2 protein fused with V5 tag was detected, and this signal was missing in un-transfected cells (Fig. 7c). The GFM2 protein level significantly increased in the transfected cells, with almost doubled SDHB (Complex II) and MTCO1 (Complex IV) protein content, compared to the un-transfected control (Fig. 7c). These data show that GFM2 upregulation could be directly causing elevated mitochondrial OXPHOS protein content in hyperglycaemic renal tubular cells.

The respiration profile showed lower OCR levels in the pRP-GFM2/V5 transfected cells, compared to the Sham- and un-transfected cells (Fig. 7d). In the pRP-GFM2/V5 transfected cells, the basal respiration and ATP-linked respiration level was significantly reduced, when compared to Sham-transfected control (Fig. 7d-e, basal respiration: 7.11 ± 0.74 vs. 5.12 ± 0.72, *p* < 0.0001; ATP-linked respiration: 5.24 ± 0.57 vs. 3.81 ± 0.63, *p* < 0.0001). Similarly, cell maximal respiration level (14.73 ± 1.66 vs. 11.65 ± 1.82, *p* < 0.001) and spare respiratory capacity (7.62 ± 0.97 vs. 6.53 ± 1.13, *p* = 0.022) was also significantly lower in pRP-GFM2/V5 transfected cells than that in Sham-transfected control. The addition of transfection reagents did not affect cellular respiration, as there was no significant difference between un-transfected and Sham-transfected groups in all the assessed parameters. Our results demonstrate that GFM2 upregulation contributes to hyperglycaemia-induced mitochondrial dysfunction in renal tubular cells in diabetes.

## Discussion

We previously reported that NSA2, a putative cytosolic ribosomal biogenesis factor (Glasgow et al. 2017), which had been shown to be involved in cell cycle regulation and suggested as a putative protooncogene, was expressed at higher levels in diabetic mouse kidneys, in renal cells exposed to diabetic conditions, and in blood samples from patients with diabetic kidney disease (Shahni et al. 2012, 2013; Wu et al. 1999; Zhang et al. 2010). However, the underlying mechanisms that contributed to NSA2 up-regulation in diabetes and diabetic kidney disease, and the impact of this on the kidney or cellular function remained unknown. In the current study, we show that *NSA2* is syntenic to *GFM2*, encoding a ribosome biogenesis factor (Paternoga et al. 2020) involved in disassembly of mitochondrial mRNA at the termination of mitochondrial protein translation (Fukumura et al. 2015). This study links the regulation of protein synthesis in two cellular compartments, the cytosol and mitochondria, and potentially provides a mechanistic link between aberrant protein synthesis, mitochondrial dysfunction and CKD.

Firstly, we demonstrate that human *NSA2* and *GFM2* are syntenic, their synteny shows very strong evolutionary conservation in multiple verterbrata species with the two genes located on the same chromosomes and almost always on opposite strands, transcribed in opposite orientations and sharing a common 5’ regulatory region (Fig. 2). This synteny has likely been conserved for ~ 400 million years since it is found in lungfish, an ancestor to mammals estimated to have appeared on Earth around 400 million years ago (Jorgensen and Joss 2010). Genes within a synteny block can share common functions, are often co-regulated, and component genes in certain metabolic gene clusters show conserved synteny and co-expression patterns (Chen et al. 2019; Kikuta et al. 2007; Liu et al. 2018). The adjacent positioning of co-regulated gene pairs is widely conserved across eukaryotes (Arnone et al. 2012). Therefore, the synteny of *NSA2* and *GFM2* in humans suggests co-regulated functions. The common 5’ non-coding region between human *NSA2* and *GFM2* shows less conservation, but the coding regions of the 2 genes are highly conserved, this conservation of the coding regions is seen even in the two species of Actinopterygii where the synteny was not conserved (Fig. 2). Such strong evolutionary conservation of *NSA2* and *GFM2* coding regions suggests they may play a fundamental functional role in eukaryotic cells, and the lack of conservation of the shared 5’ non-coding region suggests that regulatory processes of these functional roles may have diverged.

Secondly, we show, as predicted from their synteny, that the expression of *NSA2* and *GFM2* is co-regulated. Their shared 5’ non-coding region contains multiple transcription factors that may regulate both *NSA2* and *GFM2*. The Metal Response Element (MRE), located within this 381 bp region, is conserved between humans, monkeys and mice, is the cognate binding site for MTF1, which is a zinc sensing transcription factor (Balamurugan et al. 2004). In HK-2 cells grown in the presence and absence of zinc, the transcription of *NSA2* and *GFM2* mRNAs showed a dose-dependent increase upon the addition of zinc, suggesting that zinc can modulate the expression of both *NSA2* and *GFM2 in vitro* (*p < 0.05*), possibly via activation of MTF1. The expression of genes involved in common functions is often regulated by common mechanisms (Snel, 2006, Marco et al. 2009). Using human single-cell datasets, many adjacent genes were shown to be co-regulated and functionally related, with their co-regulation maintained at the protein level according to further analysis using proteomics data (Ribeiro et al. 2022). Single-cell ATAC-sequencing showed that > 95% of co-regulated gene pairs share common regulatory elements (Ribeiro et al. 2022). Our data suggesting that *NSA2* and *GFM2* may be co-regulated by common regulators is in line with these published studies. Interestingly, other genes involved in ribosome biogenesis, cell growth and development have previously been shown to be significantly enriched for adjacent gene pairs and co-regulated (Arnone et al. 2012).

Thirdly, we showed that the mRNA expression of both *NSA2* and *GFM2* is increased in renal tissue from CKD patients, and their expression was significantly correlated with each other suggesting that they may play a role in CKD. Both genes also showed high expression in human renal tubular cell clusters. This is the first demonstration of altered expression of either gene in human renal biopsies. the functional impact of the increased levels of *NSA2* and *GFM2* in CKD remain unknown.

Fourthly, we showed that both NSA2 and GFM2 mRNA/proteins were increased in human renal tubular cells grown in hyperglycaemic conditions, this increase was accompanied by alterations in the levels of several key mitochondrial proteins, part of the 5 complexes of the OXPHOS system. The glucose-induced elevated expression of the two genes did not impact total cellular protein content, but it seemed to affect specific mitochondrial subunit levels. The protein levels of selected mitochondrial OXPHOS subunits representing nuclear encoded complex I (NDUFB8), Complex II (SDHB), and Complex III (UQCRC2) were significantly increased, and Complex V (ATP5A), and mitochondrial genome encoded Complex IV (MTCO1) showed a non-significant increase. Interestingly, protein abundance of the mitochondrial OXPHOS system has been previously reported to be elevated in diabetes and diabetic complications both in vivo and in vitro (Alimujiang et al. 2020; Cleveland et al. 2020; Misu et al. 2007; Parker et al. 2021). For instance, in diabetic mouse livers, the protein abundance of the mitochondrial respiratory chain significantly increased, particularly pronounced in Complex IV in the T1DM model and in Complex I in the T2DM model (Misu et al. 2007). In liver biopsies from T2DM patients, increased OXPHOS protein content correlated with fasting hyperglycaemia (Misu et al. 2007). In primary human proximal renal tubular cells cultured in a high glucose environment, Cleveland et al. found increased protein levels of the mitochondrial OXPHOS system with reduced ATP production and oxygen consumption rate, matching the results from the current study (Cleveland et al. 2020). Whilst the increase in OXPHOS subunit levels may suggest improved mitochondrial function, it has the opposite effect as shown below.

Fifthly, we showed that in cells where we induced increased NSA2 and GFM2 mRNA/protein and increased levels of OXPHOS subunits under hyperglycaemic conditions, we saw reduced mitochondrial respiration capacity, with significant reductions in basal respiration, maximal respiration, and spare respiratory capacity. These data agree with our previous findings that hyperglycaemia can damage cellular respiration in HK-2 cells (Czajka and Malik 2016). The downregulation of mitochondrial respiratory capacity in diabetes may result from a variety of factors, such as increased production of ROS and GAPDH inhibition (Brownlee 2005; Colussi et al. 2000). Mitochondrial ribosome disorders could also be a potential reason. Karim et al. suggested in a review that in lung disease, mitochondrial ribosome disorders can cause mitochondrial respiratory defects, leading to severe clinical symptoms (Karim et al. 2022). In islets from type 2 diabetes, haploinsufficiency of *CR6-interacting factor 1 (CRIF1)*, which encodes a mitochondrial ribosome protein, is associated with reduced mitochondrial respiration (Hong et al. 2022), and in cultured hepatocytes derived from liver-specific CRIF1 knockout mice, the mitochondrial maximal respiratory capacity was significantly lower compared to the WT control (Song et al. 2022). Human ribosome-binding factor A (hsRBFA) is a mitochondrial ribosome assembly factor facilitating the transcriptional modification of 12 S rRNA (Zhou et al. 2023). Deficiency of hsRBFA has been found capable of suppressing mitochondrial protein translation and respiration (Zhou et al. 2023). Since GFM2 is associated with ribosome recycling in mitochondria, while using hyperglycaemic cells, we could not determine whether the loss in cellular respiration capacity and the increased OXPHOS protein levels were directly caused by GFM2 up-regulation or some aspect of hyperglycaemia induced changes that we had not measured.

Sixthly, we showed that inducing over expression of GFM2 in the absence of hyperglycaemia also caused similar changes to those under hyperglycaemic conditions, providing support for the view that GFM2 over expression rather than hyperglycaemia per se may be causative of mitochondrial dysfunction. Using transient transfection of GFM2, increased GFM2 expression resulted in increases of some OXPHOS subunits and in parallel, reduced cellular respiration. Among the examined proteins, the increase of nuclear-genome encoded mitochondrial SDHB and mitochondrial-genome encoded MTCO1 protein was seen in the cells transfected with GFM2 expressing plasmids, pRP-GFM2/V5, suggesting that GFM2 upregulation in diabetic tubular cells directly causes the changes. The respiratory profile of tubular cells showed that the OCR levels of mitochondrial respiration parameters were negatively affected by exposure to high glucose, and this study noted for the first time that the upregulation of GFM2 may contribute to this mitochondrial dysfunction, as decreased OCR in mitochondrial respiration parameters were also observed in pRP-GFM2/V5 transfected cells.

Taken together, our data suggest that increased expression of mitochondrial GFM2 and cytosolic NSA2 may contribute to ribosome dysfunction in mitochondria and cytosol. The reduction of mitochondrial respiration parameters with increased OXPHOS proteins caused by GFM2 upregulation suggests that in diabetic renal cells, hyperglycaemia-induced mitochondrial dysfunction may result from dysregulated ribosome functions in cytosol and mitochondria. Whilst we have demonstrated mitochondrial dysfunction, we have not directly shown cytosolic ribosomal dysfunction. Our study has some limitations. Our proposal that NSA2 and GFM2 are co-regulated is based on their conserved 5’ regulatory regions, their expression patterns in human tissue and experimental cell models, their regulation by MTF1, located in the common 381 bp untranslated region, and the evidence from multiple other studies showing co-regulation of genes that display such synteny. To prove the co-regulation of the two genes requires promoter dissection and/or DNA foot-printing. Another limitation was in our transfection experiment where we found that the increase in recombinant GFM2 protein levels was modest despite a high increase in mRNA levels. It is likely that we did not achieve optimal translation of the recombinant GFM2, which increased by 37% (compared to the 42% increase of the endogenous protein seen under high glucose). The reasons for the modest increase remain unclear, however, it is possible that increase in protein levels may be detrimental since in our experiments longer transfection times were lethal to the cells. Another possibility is that since the native expression level of GFM2 protein is already quite high in human kidneys, according to the Human Protein Atlas Database (Uhlén et al. 2005), increasing levels further may be blocked through some unknown regulatory mechanisms. Therefore, the mechanisms controlling upregulation of GFM2 protein need further investigation. Our study was limited to the impact of GFM2 overexpression. In future studies, knockdown of *NSA2* and/or *GFM2* genes to evaluate the functional impact on protein synthesis and mitochondrial OXPHOS should be carried out. A final limitation is that the direction causality of elevated GFM2 and mitochondrial dysfunction needs confirmation, as this would provide an important step in the pathway of renal disease. It is possible that early stages of mitochondrial dysfunction trigger a protective response, leading to an increased demand of proteins involved in damage repairing or mitochondrial OXPHOS, thereby enhancing the elevation of NSA2 and GFM2 expression. Alternatively, it maybe that oxidative stress or related pathways resulting from hyperglycemia initiate the change in GFM2 which then is one of several mechanisms contributing to mitochondrial dysfunction.

## Conclusions

Our data link the regulation of 2 highly conserved genes, *NSA2* and *GFM2*, connected to ribosomes in two different cellular compartments, cytosol and mitochondria, to kidney disease and shows that their dysregulation may be involved in mitochondrial dysfunction. *GFM2* was discovered syntenic to *NSA2*, with both genes sharing 5’ regulatory regions. Their shared 5’ regulatory region suggests their co-regulation which was demonstrated in tubular cells in vitro and in human kidneys from CKD patients. The demonstration that GFM2 upregulation achieved by transfection increased certain OXPHOS complexes and reduced mitochondrial respiration suggests that in diabetes, the detrimental effect on mitochondrial function could be directly caused by the increase of GFM2.

## Electronic supplementary material

Below is the link to the electronic supplementary material.

Supplementary Material 1

Supplementary Material 2

Supplementary Material 3

Supplementary Material 4

## Data Availability

The datasets generated and/or analysed during the current study are available in the Kidney Precision Medicine Project dataset (https://www.kpmp.org) and Nakagawa CKD Kidney dataset (http://www.ncbi.nlm.nih.gov/geo/query/acc.cgi?acc=GSE66494).

## Abbreviations

CKD: Chronic kidney disease
DMEM: Dulbecco’s modified eagle’s medium
ELK1: ETS Like-1
ETC: Electron transport chain
FCCP: Carbonyl cyanide-4 (trifluoromethoxy) phenylhydrazone
GABP-B: GA Binding Protein Transcription Factor Subunit Beta
GFM2: GTP-dependent ribosome recycling factor mitochondrial 2
HC: Healthy control
HCL-G1: Hairy cell leukaemia gene 1
HES1: Hes Family BHLH Transcription Factor 1
HG: High glucose:20mM glucose
KAISO: Zinc Finger and BTB Domain Containing 33
MRE: Metal Response Element
MTF1: Metal Regulatory Transcription Factor 1
mtUPR: Mitochondrial unfolded protein response
NG: Normal glucose:5mM glucose
NSA2: Nop-7-associated 2
OCR: oxygen respiration rate
PAX5: Paired Box Gene 5
PCR: Polymerase chain reaction
SMAD4: Mothers against decapentaplegic homolog 4
TFBS: Transcriptional factor binding sites
