# Supplementary material for: Co-regulation and synteny of GFM2 and NSA2 links ribosomal function in mitochondria and the cytosol with chronic kidney disease
Source: Mol Med. 2024 Oct 13;30:176. doi: 10.1186/s10020-024-00930-8 (PMC11476648; doi:10.1186/s10020-024-00930-8)
Supplement: Supplementary file 1 — Supplementary Material 1 [file 10020_2024_930_MOESM1_ESM.docx]

**Additional file 1.**

Title: Detail information of the potential transcription factors of human *NSA2* gene

| **TF^1^** | **Position (nt)^2^** | **Details related to NSA2** | **Published NSA2 functions** | **Reference /PMID link** |
| --- | --- | --- | --- | --- |
| PAX5 | -36 to -9 | - Diseases associated with PAX5 include Leukaemia | - NSA2 is a tumour suppressor gene and mutations can cause human-hairy cell leukaemia. | Wu et al, 1999. [PMID 10486207](https://pubmed.ncbi.nlm.nih.gov/10486207/)  Mohammad et al.2015.PMID: [25428382](https://pubmed.ncbi.nlm.nih.gov/25428382/)  Jia, et al. 2022, PMID: [36387144](https://pubmed.ncbi.nlm.nih.gov/36387144) |
| HES1_Q2 | -84 to -70 | - Mutations is related to acute lymphoblastic leukaemia |  | Kato, T et al. 2015. PMID: [25234168](https://pubmed.ncbi.nlm.nih.gov/25234168/) |
| MTF1_Q4 | -136 to -127 | - induce expression of metallothionein and other genes involved in metal homeostasis in pancreatic Beta Cell Pathophysiology | - Biomarker of diabetic kidney disease | Mohammed et al.2021. PMID: [33652748](https://pubmed.ncbi.nlm.nih.gov/33652748) |
| KAISO | -211 to -202 | - regulate p53 pathway - subcellular partitioning is associated with breast cancer progression | - G1/S transition - p53 and p21 pathways | Zhang et al, 2010. [PMID: 19932687](https://pubmed.ncbi.nlm.nih.gov/19932687/)  Li et al, 2013. [PMID: 23912275](https://pubmed.ncbi.nlm.nih.gov/23912275/)  Koh et al.2014. PMID: [25288747](https://pubmed.ncbi.nlm.nih.gov/25288747)  Sandeep et al.2021. PMID: [33526872](https://pubmed.ncbi.nlm.nih.gov/33526872) |
| ELK1 | -240 to -227 | - a nuclear target for the ras-raf-MAPK signalling cascade | - Nuclear localization signals (NLSs) at N terminal - Ribosomal biogenesis and protein synthesis | Cristina et al. 2007. PMID: [17962350](https://pubmed.ncbi.nlm.nih.gov/17962350/) |
| GABP_B | -241 to -230 | - also known as nuclear respiratory factor 2 - be involved in the activation of cytochrome oxidase expression and nuclear control of mitochondrial function - regulates essential trans-acting factors in 60s ribosome biogenesis |  | Yang et al. 2014. PMID: [24958105](https://pubmed.ncbi.nlm.nih.gov/24958105/)  Xing et al, 2018. [PMID: 30243719](https://pubmed.ncbi.nlm.nih.gov/30243719/)  Alessandra et al.2006. PMID: [16530192](https://pubmed.ncbi.nlm.nih.gov/16530192/) |
| Smad4 | -331 to -323 | - Smad proteins are phosphorylated and activated by transmembrane serine-threonine receptor kinases in response to transforming growth factor (TGF)-β signaling | - NSA2 is TGFβ1 inducible protein - Biomarker of diabetic kidney disease | R Shahni et al, 2012. PMID: [22095236](https://pubmed.ncbi.nlm.nih.gov/22095236/)  R Shahni et al, 2013. [PMID: 23220173](https://pubmed.ncbi.nlm.nih.gov/23220173/)  Tsuchida et al.2003. PMID: [12753287](https://pubmed.ncbi.nlm.nih.gov/12753287/) |

^1^TF, transcription factor

^2^The NSA2 start codon is position +1. Minus (-) means the position is upstream of the start codon.
