# Supplementary material for: Co-regulation and synteny of GFM2 and NSA2 links ribosomal function in mitochondria and the cytosol with chronic kidney disease
Source: Mol Med. 2024 Oct 13;30:176. doi: 10.1186/s10020-024-00930-8 (PMC11476648; doi:10.1186/s10020-024-00930-8)
Supplement: Supplementary file 2 — Supplementary Material 2 [file 10020_2024_930_MOESM2_ESM.docx]

**Additional file 2.** *NSA2* and *GFM2* mRNA expression levels in HK-2 cells grown in media with and without ZnSO4.

|  | NoZn | 20μMZn | 40μMZn |
| --- | --- | --- | --- |
| NSA2 | 1.57($\pm0.47)$ | 2.54($\pm0.08)$ | 2.73($\pm0.30)$ |
| GFM2 | 25.16($\pm1.64)$ | 36.31($\pm4.69)$ | 39.14($\pm3.42)$ |

**Legend:** The *NSA2* and *GFM2* mRNA expression levels were calculated as mRNA copy numbers relative to per 1000 *GAPDH.* The fold changes were normalized to the mRNA expression level in NoZn media. The table shows the mean levels ( $\pm$standard deviation) of *NSA2* and *GFM2* mRNA expression levels in each group. n=3. The data were analysed using one-way ANOVA. **p<0.05, ** p<0.01.*
