# Supplementary material for: Co-regulation and synteny of GFM2 and NSA2 links ribosomal function in mitochondria and the cytosol with chronic kidney disease
Source: Mol Med. 2024 Oct 13;30:176. doi: 10.1186/s10020-024-00930-8 (PMC11476648; doi:10.1186/s10020-024-00930-8)
Supplement: Supplementary file 3 — Supplementary Material 3 [file 10020_2024_930_MOESM3_ESM.docx]

**Additional file 3.** Reference Uniform Manifold Approximation and Projection of kidney cell clusters from KPMP single cell RNA-Seq.


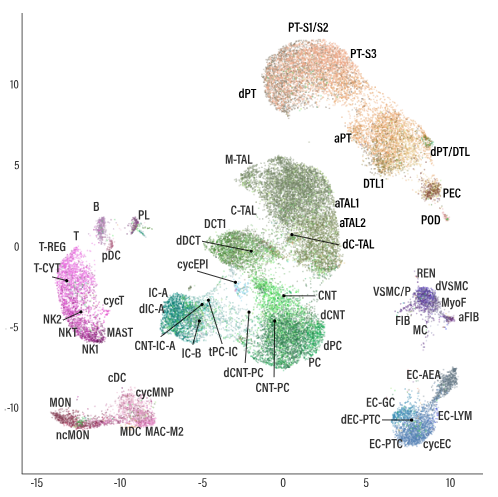


**Legend:** The Uniform Manifold Approximation and Projection (UMAP) was generated from KPMP tissue atlas. The abbreviation of cell clusters and subtypes are labelled. The full names of the abbreviations are listed in Addition file 4.
