# Supplementary material for: Co-regulation and synteny of GFM2 and NSA2 links ribosomal function in mitochondria and the cytosol with chronic kidney disease
Source: Mol Med. 2024 Oct 13;30:176. doi: 10.1186/s10020-024-00930-8 (PMC11476648; doi:10.1186/s10020-024-00930-8)
Supplement: Supplementary file 4 — Supplementary Material 4 [file 10020_2024_930_MOESM4_ESM.docx]

**Additional file 4.** The abbreviation of clusters and subtypes in KPMP single cell RNA-Seq.

| Abbreviation | Full Name |
| --- | --- |
| cycEC | Endothelial Cell (cycling2) |
| EC-AEA | Afferent / Efferent Arteriole Endothelial Cell |
| B | B Cell |
| cDC | Classical Dendritic Cell |
| CNT | Connecting Tubule Cell |
| dCNT | Connecting Tubule Cell (degenerative3) |
| CNT-IC-A | Connecting Tubule Intercalated Cell Type A |
| CNT-PC | Connecting Tubule Principal Cell |
| dCNT-PC | Connecting Tubule Principal Cell (degenerative3) |
| C-TAL | Cortical Thick Ascending Limb Cell |
| dC-TAL | Cortical Thick Ascending Limb Cell (degenerative3) |
| T-CYT | Cytotoxic T Cell |
| DTL1 | Descending Thin Limb Cell Type 1 |
| dDCT | Distal Convoluted Tubule Cell (degenerative3) |
| DCT1 | Distal Convoluted Tubule Cell Type 1 |
| cycEPI | Epithelial Cell (cycling2) |
| FIB | Fibroblast |
| aFIB | Fibroblast (maladaptive1) |
| EC-GC | Glomerular Capillary Endothelial Cell |
| IC-A | Intercalated Cell Type A |
| dIC-A | Intercalated Cell Type A (degenerative3) |
| IC-B | Intercalated Cell Type B |
| EC-LYM | Lymphatic Endothelial Cell |
| MAC-M2 | M2-Macrophage |
| MAST | Mast Cell |
| M-TAL | Medullary Thick Ascending Limb Cell |
| MC | Mesangial Cell |
| MON | Monocyte |
| MDC | Monocyte-derived Cell |
| cycMNP | Mononuclear Phagocyte (cycling2) |
| MyoF | Myofibroblast |
| NK1 | Natural Killer Cell Type 1 |
| NK2 | Natural Killer Cell Type 2 |
| NKT | Natural Killer T Cell |
| ncMON | Non-classical Monocyte |
| PEC | Parietal Epithelial Cell |
| EC-PTC | Peritubular Capillary Endothelial Cell |
| dEC-PTC | Peritubular Capillary Endothelial Cell (degenerative3) |
| PL | Plasma Cell |
| pDC | Plasmacytoid Dendritic Cell |
| POD | Podocyte |
| PC | Principal Cell |
| dPC | Principal Cell (degenerative3) |
| tPC-IC | Principal-Intercalated Cell (transitional4) |
| aPT | Proximal Tubule Epithelial Cell (repairing1) |
| dPT | Proximal Tubule Epithelial Cell (degenerative3) |
| dPT/DTL | Proximal Tubule Epithelial Cell / Descending Thin Limb Cell (degenerative3) |
| PT-S1/S2 | Proximal Tubule Epithelial Cell Segment 1 / Segment 2 |
| PT-S3 | Proximal Tubule Epithelial Cell Segment 3 |
| T-REG | Regulatory T Cell |
| REN | Renin-positive Juxtaglomerular Granular Cell |
| T | T Cell |
| cycT | T Cell (cycling2) |
| aTAL1 | Thick Ascending Limb Cell Cluster 1 (repairing1) |
| aTAL2 | Thick Ascending Limb Cell Cluster 2 (repairing1) |
| dVSMC | Vascular Smooth Muscle Cell (degenerative3) |
| VSMC/P | Vascular Smooth Muscle Cell / Pericyte |

^1^Adaptive/maladaptive/repairing represented by cells that retain differentiation markers of reference states, albeit at lower levels, but also show expression of known injury associated genes, mesenchymal markers or factors promoting inflammation or fibrosis.

^2^cycling represented by enrichment of cell cycle genes.

^3^degenerative marked loss of differentiation markers, and/or increased %ERT, %MT, and/or marked decrease in genes detected.

^4^transitional represented by an intermediate state showing markers of cells sharing the same parental lineage.
